# New Insight on 2D In Vitro Angiogenesis Models: All That Stretches Is Not a Tube

**DOI:** 10.3390/cells11203278

**Published:** 2022-10-18

**Authors:** Irina Beloglazova, Ekaterina Zubkova, Konstantin Dergilev, Yulia Goltseva, Yelena Parfyonova

**Affiliations:** 1 Laboratory of Angiogenesis, Chazov National Medical Research Center of Cardiology, Moscow 121552, Russia; 2 Department of Biochemistry and Molecular Medicine, Faculty of Medicine, Lomonosov Moscow State University, Moscow 119192, Russia

**Keywords:** angiogenesis, co-culture, Notch, ephrins, extracellular matrix, EndMT, tip cell, MSC, endothelial cells, Matrigel

## Abstract

**Highlights:**

- Tube formation on Matrigel^TM^ and tube formation in co-culture with MSCs are two different stages of angiogenesis.
- uPA, uPAR, Jagged1, and Notch2 are common upregulated genes for ECs on Matrigel^TM^, in co-culture and in dividing/migrating cells.
- EndMT activated at a much greater extent in ECs in a co-culture model than in a Matrigel^TM^ assay.
- Only in the Matrigel^TM^ assay are the Notch and Hippo pathway-related genes upregulated.

**Abstract:**

A Matrigel-based tube formation assay is a simple and widely accepted 2D angiogenesis model in vitro. Extracellular matrix (EM) proteins and growth factors (GFs) from Matrigel^TM^ exclusively trigger endothelial cell (EC) tubular network (ETN) formation. Co-culture of ECs with mesenchymal stromal cells (MSCs) is another and more reliable in vitro angiogenesis assay. MSCs modulate ETN formation through intercellular interactions and as a supplier of EM and GFs. The aim of the present study was to compare the expression profile of ECs in both models. We revealed upregulation of the uPA, uPAR, Jagged1, and Notch2 genes in dividing/migrating ECs and for ECs in both experimental models at 19 h. The expression of endothelial–mesenchymal transition genes largely increased in co-cultured ECs whereas Notch and Hippo signaling pathway genes were upregulated in ECs on Matrigel^TM^. We showed that in the co-culture model, basement membrane (BM) deposition is limited only to cell-to-cell contacts in contrast to Matrigel^TM^, which represents by itself fully pre-assembled BM matrix. We suggest that ETN in a co-culture model is still in a dynamic process due to immature BM whereas ECs in the Matrigel^TM^ assay seem to be at the final stage of ETN formation.

## 1. Introduction

Endothelium in healthy organisms is a cellular monolayer of tightly contacted cells. Various external influences such as hypoxia, tissue damage, or inflammatory factor release could cause the disruption of tight cell contacts and a subsequent increase in vascular permeability and initiation of angiogenesis, the regrowth of new blood vessels from already existing ones. While new vessels grow, endothelial cells communicate with mural cells and with the extracellular matrix (EM), remodeling it. 

Although much is already known about angiogenesis, there are still a lot of blank spots, which researchers are trying to explore in different in vitro models.

The Matrigel^TM^ model of angiogenesis [1] is the best-known and simplest 2D in vitro model. It evaluates cells for their ability to form a tubular network (ETN) through interaction with growth factors (IGF-1, TGF-b, EGF, PDGF, bFGF, NGF, VEGF, and others) and EM proteins (~60% laminin, ~30% collagen IV, and ~8% entactin and perlecan) included in Matrigel^TM^ [2]. 

The composition of Matrigel^TM^ has much in common with basement membrane (BM), the core protein components of which are laminins, collagen IV, nidogens, and the heparan sulfate proteoglycans perlecan and agrin [3].

Another 2D model of angiogenesis in vitro is the co-culturing of ECs with stabilizing cells: mesenchymal stromal cells (MSCs) on plastic that are not covered with EM proteins. In this model, the sources of EM and growth factors (GFs) are MSCs [4]. MSCs build a fibronectin scaffold that further initiates basement membrane assembly [4]. In addition, the intercellular interactions between ECs and MSCs themselves contribute to the formation of ETN [4,5].

In this work, we attempt to separate the contribution of EM and intracellular contacts to ETN formation. We compared the expression profiles of an angiogenesis-associated gene set in ECs in different models: Ecs forming ETN on Matrigel^TM^, Ecs in co-culture with MSCs, Ecs in a confluent monolayer, and dividing/migrating ECs.

## 2. Materials and Methods

### 2.1. Cell Isolation and Culture

The MSC cell line ASC52Telo was obtained from the collection of human biomaterials of the Institute for Regenerative Medicine (Lomonosov Moscow State University, collection ID: MSU_MSC_AD; repository catalogue at www.human.depo.msu.ru). MSCs were cultured in DMEM-GlutaMAX™ (ThermoFisher Scientific, Waltham, MA, USA) with 10% fetal bovine serum (FBS) (Cytiva, Marlborough, MA, USA) and penicillin/streptomycin (ThermoFisher Scientific, Waltham, MA, USA). Umbilical cords from healthy donors were collected in the Obstetric Department of the V. I. Kulakov National Medical Research Center for Obstetrics, Gynecology, and Perinatology after written informed consent was obtained from all women. Endothelial cells from the umbilical vein (HUVECs) were isolated as previously described [6] and cultured in complete EGM-2 medium (Lonza, Basel, Swiss). MSCs and HUVECs were grown until 70% confluence in a humidified chamber incubator at 37 °C and 5% CO_2_. Before the co-culture experiments, cells were detached with 0.05% trypsin/EDTA (ThermoFisher Scientific, Waltham, MA, USA). In all experiments, HUVECs were used at passage 3 to 5. In each experiment, we used at least three biological repeats (one donor per repeat).

### 2.2. Tube Formation Assay on Matrigel^TM^ In Vitro

The in vitro endothelial cell tube formation assay was performed and quantified as described by us previously [7]. Briefly, HUVECs were seeded onto the 60 cm^2^ Petri dish covered with a thick layer of Matrigel^TM^ (Corning, NY, USA) at a density of 5 × 10^4^ cells/cm^2^ and cultured for 19 h in the EGM-2 media (Lonza, Basel, Swiss). Cells were utilized for mRNA expression analysis or fixed with 4% formaldehyde and visualized using a wide-field fluorescent Axiovert 200 M Microscope (Zeiss, Oberkochen, Germany).

### 2.3. HUVEC-MSC Two-Dimensional (2D) Co-Culture Model

A co-culture model was performed as described by us previously [4]. Briefly, the mixtures of HUVEC:MSC at a ratio of 1:3 and at a total density of 6 × 10^4^ cells/cm^2^ were seeded onto a 100 cm^2^ Petri dish and co-cultured for 19 h in EGM-2 for mRNA expression analysis or for 48 h for immunofluorescent staining.

### 2.4. The 2D Co-Culture Model of HUVECs with MSCs and Its 48 h Synthesized Extracellular Matrix (EM)

MSCs were grown as a monoculture for 48 h (“48 hold MSCs”) to allow synthesis/secretion/assembly of extracellular matrix. HUVECs were then seeded on the top of “48-h-old MSCs” and co-cultured for an additional 19 h in EGM-2 for mRNA expression analysis.

### 2.5. 4 Condition of HUVECs Growth

HUVECs were seeded in four growth conditions:

(1) Onto a 60 cm^2^ Petri dish covered with Matrigel^TM^ at a density of 5 × 10^4^ cells/cm^2^.
(2) As a co-culture with MSCs at a total cell density of 6 × 10^4^ cells/cm^2^ at a ratio of 1:3 of HUVEC: MSC.
(3) As a “proliferating/motile” sparsely populated monoculture at a density 3.6 × 10^4^ cells/cm^2^ on a 60 cm^2^ Petri dish.
(4) As a “static monolayer” densely populated monoculture at a density of 7 × 10^4^ cells/cm^2^ on a 100 cm^2^ Petri dish.

HUVECs were cultivated for 19 h in EGM-2.

### 2.6. Immunofluorescent Staining

Monocultures of HUVECs and MSC-HUVEC co-cultures were grown on glass coverslips for 48 h or on Matrigel^TM^ for 19 h. HUVECs were pre-loaded with 5 μM CellTracker ™ Green CMFDA fluorescent dye (ThermoFisher Scientific, Waltham, MA, USA) for 40 min according to the manufacturer’s protocol. Cells were fixed with 4% formaldehyde and probed with the following primary antibodies (Abs): anti-CD31 (Biolegend, San Diego, CA, USA), anti-laminin (Abcam), anti-collagen 1 (Abcam), anti-collagen 4 (Abcam, Cambridge, UK), anti-fibronectin (Abcam, Cambridge, UK), or isotype-matched control immunoglobulins, followed by fluorophore-conjugated secondary Abs: goat anti-rabbit Alexa Fluor 594 (Invitrogen) or goat anti-mouse Alexa Fluor 594 (Invitrogen). Stained cells were visualized using a wide-field fluorescent Axiovert 200 M Microscope (Zeiss, Oberkochen, Germany).

### 2.7. RNA Isolation, Reverse Transcription, and Real-Time Quantitative PCR

HUVECs were grown for mRNA expression analysis on Matrigel^TM^, in co-culture with MSCs, as a sparsely populated monoculture (3.6 × 10^4^ cells/cm^2^) or densely populated (7 × 10^4^ cells/cm^2^) for 19 h in EGM-2. Cells were detached with trypsin/EDTA and washed free of trypsin. Cells from co-cultures were separated using a flow cell sorter MoFlo (Agilent, Santa Clara, CA, USA) as described by us previously [4].

Total RNA was isolated from MSCs or HUVECs either grown in monoculture or separated from co-culture using a RNeasy Mini Kit (QIAGEN, Germantown, MD, USA). First-strand cDNA was synthesized with random hexamer primers using a RevertAidTM First Strand cDNA Synthesis Kit (ThermoFisher Scientific, Waltham, MA, USA). Real-time PCR was performed with SYBR Green intercalating dye (Syntol) using a StepOnePlusTM Real-Time PCR System (ThermoFisher Scientific, Waltham, MA, USA); the primers used for PCR are listed in Table 1. Reaction mixtures (25 μL) contained 1–5 ng of cDNA, 10 pmol of each primer, 10 μL of dNTP/DNA polymerase solution (Sintol, Moscow, Russian Federation), and deionized water up to 25 μL. Control mixtures contained all components except the cDNA template, which was replaced by deionized water. After denaturation (95 °C, 10 min), 40 amplification cycles were performed for all primer pairs, with annealing/elongation at 60 °C for 60 s. The specificity of amplification was analyzed by the melting stage upon PCR completion.

### 2.8. Data Analysis

The mRNA expression data were analyzed using the online program Heatmapper [8]. The data are presented in three repeats. Clustering Method: Average linkage. Distance Measurement Method: Pearson.

Functional protein association network analysis was performed in STRING [9].

### 2.9. Statistical Analysis

Data are expressed as the mean ± standard deviation (SD). The statistical significance of the difference between values was determined using Student’s *t*-tests. *p* < 0.05 was considered significant.

## 3. Results

In this work, we compared the classical Matrigel-based tube formation assay [1] and 2D co-culture model [4]. 

**Figure 1:**
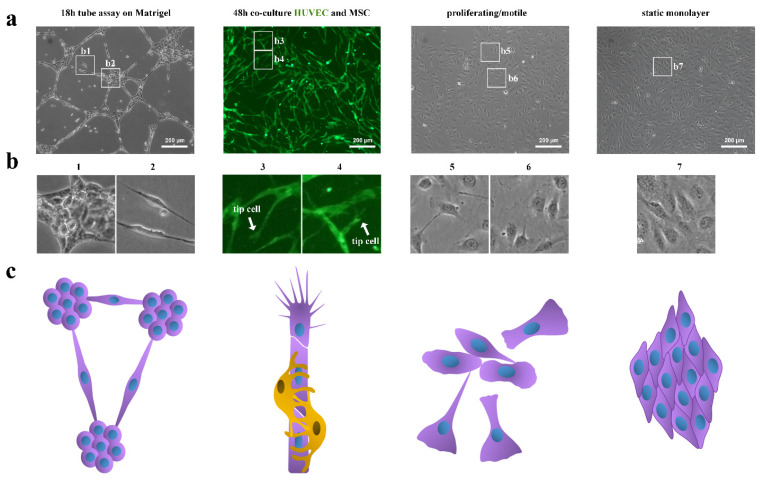
Representative images of ECs under different experimental settings. Tube assay on Matrigel^TM^ (19 h). HUVECs were seeded on Matrigel^TM^ at a density of 5 × 10^4^ cells/cm^2^ and cultured for 19 h in EGM-2 (Lonza). Co-culture model. HUVECs were preliminarily labeled with vital fluorescent dye CMFDA (green). The mixtures of HUVEC:MSC at a ratio of 1:3 and a density of 6 × 10^4^ cells/cm^2^ were seeded onto a Petri dish without matrix and co-cultured in EGM-2 (Lonza) for 48 h. Proliferating/motile culture of HUVECs: sparsely populated monoculture (3.6 × 10^4^ cells/cm^2^) in EGM-2 (Lonza) for 19 h. Static monolayer of densely populated HUVECs (7 × 10^4^ cells/cm^2^) for 19 h in EGM-2 (Lonza). Images were taken using the Axiovert 200 M Microscope (Zeiss, Oberkochen, Germany): 10× amplification (**a**), 68× amplification (**b**) of selected areas, where white arrows indicate tip cells among others. (**c**) A schematic representation of the formed cellular structures.

Endothelial cells were able to form tubular-like structures on Matrigel^TM^ (Figure 1a). At 16 h, HUVECs on Matrigel^TM^ already form tubular-like structures but after 24 h, these structures become unstable and disintegrate [10].

However, HUVECs formed tubes on Matrigel^TM^ (Figure 1a) and in co-culture with MSCs (Figure 1a) and these tubes differed in their form. HUVECs on Matrigel^TM^ formed clusters of cells with a round shape (Figure 1b1) connected by “tubes” (Figure 1b2). HUVECs in co-culture with MSCs formed a tubular network with “tip”-like cells (Figure 1b3,b4). HUVEC tubes in co-culture were in a looser state compared to the highly strained tubes on Matrigel^TM^. Cellular clusters on Matrigel^TM^ (Figure 1b1) were in a more compact form compared to the HUVEC static monolayer (Figure 1b7). Proliferating/motile HUVECs in spare culture also formed some “tubes” (Figure 1b5) and occupied more space (Figure 1b6) compared to the static monolayer (Figure 1b7).

Previously, we have shown that endothelial cells in co-culture with MSCs formed tubular-like structures that became evident by 14 h [4]. By 48 h of co-culturing with MSCs, HUVECs organized into an extensive capillary network (Figure 1a). MSCs contributed to HUVEC tube formation by the synthesis and assembly of the extracellular matrix. By 48 h of pre-culturing alone, MSCs created an organized extracellular matrix. This matrix increased HUVEC tube formation by more than 6-fold in 24 h in comparison with the co-culture of HUVECs and MSCs without pre-cultivation [4].

We chose 19 h as the optimal time point.

**Figure 2:**
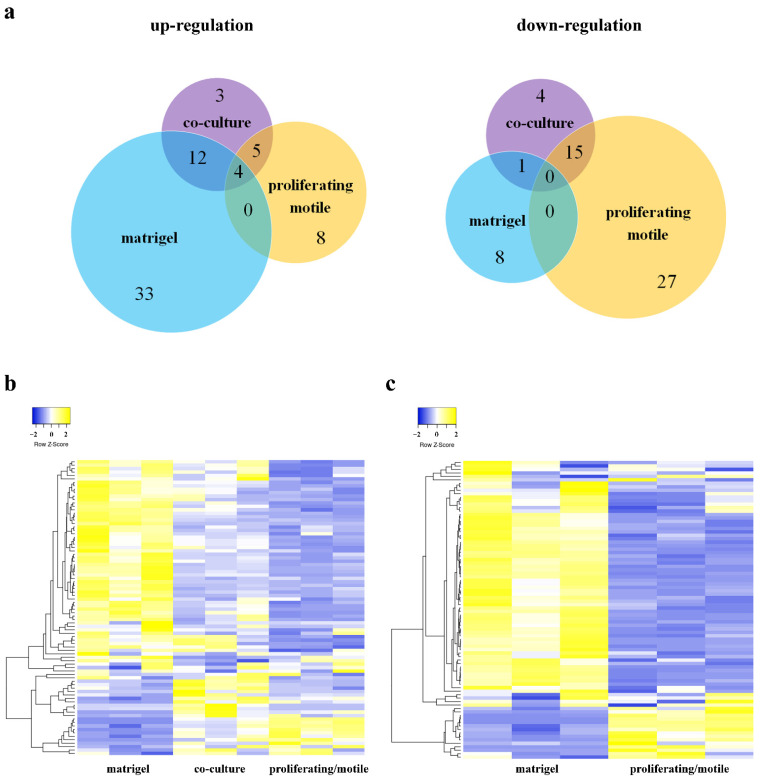
(**a**) Venn diagram summarizes the number of significantly changed genes (*p* < 0.05) that are differentially upregulated or downregulated between experimental groups compared to the HUVEC static monolayer. ECs from direct co-culture were separated using anti CD31 magnetic beads. Total RNA was isolated and specific mRNA levels were quantified by qRT PCR as described in “Methods”. (**b**) Hierarchical clustering dendrograms of differentially expressed mRNA expression patterns in HUVECs on Matrigel^TM^, in co-culture with MSCs, and proliferating/motile HUVECs analyzed using online software Heatmapper [8]. The data are presented in three repeats. Clustering Method: Average linkage. Distance Measurement Method: Pearson. (**c**) Hierarchical clustering dendrograms of differentially expressed mRNA expression patterns in HUVECs on Matrigel^TM^ and proliferating/motile HUVECs analyzed using the online software Heatmapper [8]. The data are presented in three repeats. Clustering Method: Average linkage. Distance Measurement Method: Pearson.

We analyzed the mRNA expression of endothelial gene markers (Table 2) related to angiogenesis at this time point for all culture conditions: (1) HUVECs on Matrigel^TM^, (2) HUVECs in co-culture with MSCs, (3) sparsely populated monoculture “proliferating/motile”, and (4) densely populated monoculture “static monolayer”. We found that HUVECs on Matrigel^TM^, HUVECs from co-culture, and proliferating/motile HUVECs (sparse culture) had four common reliably (compared to monolayer HUVECs) upregulating genes (Figure 2a): PLAU (uPA), PLAUR (uPAR), JAG1 (Table 1. ligand Jagged1), and NOTCH2 (Notch receptor Notch2) and no common reliably downregulated genes (compared to monolayer HUVECs).

Hierarchical cluster analysis revealed that HUVECs from co-culture and proliferating/motile HUVECs had a similar pattern of gene expression in contrast to HUVECs on Matrigel^TM^ (Figure 2b). Moreover, HUVECs on Matrigel^TM^ and proliferating/motile HUVECs had a direct opposite pattern of gene expression (Figure 2c). This means that cells in these conditions undergo different processes. This data poses many questions and led us to a more precise analysis of groups of markers attributed to different processes engaged in angiogenesis.

### 3.1. Endothelium Activation

**Figure 3:**
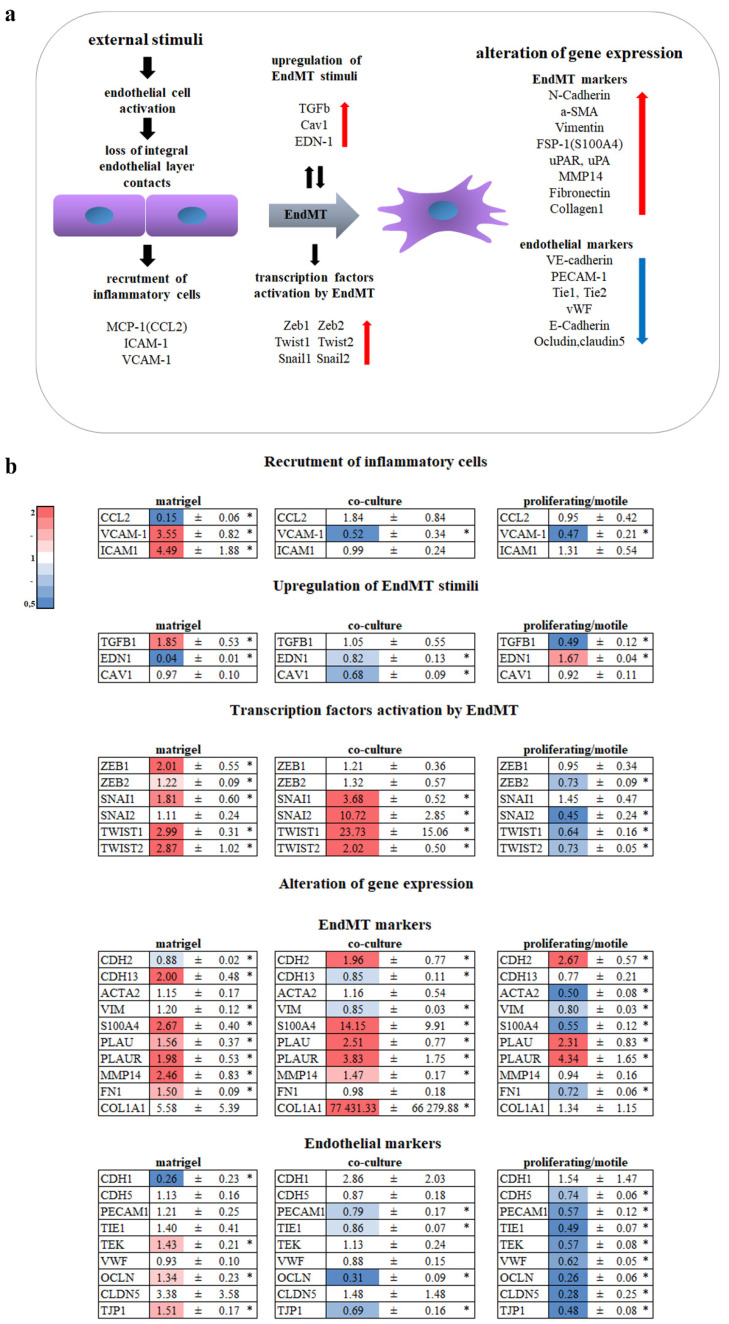
Analysis of EndMT genes. (**a**) Scheme summarizing key genes involved in EndMT and analyzed in our research. Red arrows indicate upregulated genes and blue arrows indicate downregulation of expression. (**b**) Fold changes in the mRNA levels in HUVECs from the Matrigel^TM^ assay, co-culture with MSCs, and proliferating/motile HUVECs compared to the static confluent monolayer. Cells in direct co-culture were separated using anti CD31 magnetic beads after 19 h. Total RNA was isolated and specific mRNA levels were quantified by qRT PCR as described in “Methods”. The data are presented as fold changes in the mRNA levels of cells on Matrigel^TM^, in co-culture with MSCs, and proliferating/motile HUVECs compared to the mRNA levels for the same transcript in the confluent monolayer, “*”: *p* < 0.05, *n* ≥ 3.

External stimuli such as infection, hypoxia, or injury lead to “activation” of the endothelium [11] (Figure 3a). This activation leads to the recruitment of inflammatory cells by upregulation of vascular cell adhesion molecule 1 (VCAM1), intercellular adhesion molecule 1 (ICAM1), and monocyte chemoattractant protein 1 (MCP-1; also known as CCL2). 

We can see that HUVECs on Matrigel^TM^ had an “activation” mode with VCAM-1 and ICAM-1 expression upregulation in contrast to HUVECs from co-culture and proliferating/motile HUVECs that had downregulated VCAM1 (Figure 3b). CCL2 expression was significantly downregulated in HUVECs on MSCs.

One of the angiogenic stimuli produced by ECs themselves is angiopoetin 2 (ANGPT2). ANGPT2 was upregulated only in HUVECs on Matrigel^TM^ (Figure 3b). 

### 3.2. EndMT 

Depending on the stimuli, endothelium activation may lead to the initiation of angiogenesis: cells lose intercellular contacts and become more motile. During this process, cells exhibit endothelial-to-mesenchymal transition (EndMT). The key transcriptional regulators of EndMT are Snail (SNAI1), Slug (SNAI2), Zeb1 (ZEB1), Zeb2 (ZEB2), Twist1 (TWIST1), and Twist2 (TWIST2) [12] (Figure 3a). 

Figure 3b demonstrates the upregulation of these genes in HUVECs from co-culture and in HUVECs in the Matrigel^TM^ assay. Surprisingly, in proliferating/motile HUVECs, these genes were downregulated (Figure 3b).

The loosening of intercellular contacts leads to actin rearrangement and upregulation of TGFb, EDN-1, and CAV1, proteins associated with EndMT, thus activating a positive feedback loop. Remarkably, EDN1 was downregulated up to 25-fold in HUVECs in the Matrigel^TM^ assay.

EndMT leads to alteration of gene expression: cells lose their endothelial markers (CDH1, CDH5, PECAM1, TIE1, TEK, VWF, OCLN, CLDN5, TJP1) and obtain mesenchymal ones (CDH2,CDH13, ACTA2, VIM, S100A4, PLAU, PLAUR, MMP14, FN1, COL1A1) [13,14] (Figure 3a).

We did not observe a clear picture of the gene expression pattern in all three culture conditions. We can only talk about partial EndMT. However, ECs in co-culture are involved in EndMT to a higher extent. 

### 3.3. Tip Cell

During sprouting angiogenesis, the leading (tip) cell of the growing vessel changes its morphology and undergoes partial EndMT, which enables the tip cell to be more mobile and quickly respond to external stimuli.

**Figure 4:**
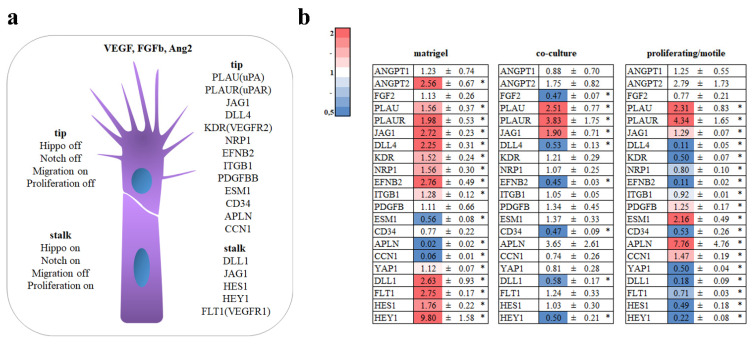
Analysis of tip and stalk genes. (**a**) Scheme summarizing tip and stalk genes in a growing vessel. (**b**) HUVECs were grown on Matrigel^TM^, in co-culture with MSCs, or as proliferating/motile HUVECs or a confluent monolayer for 19 h. Cells in direct co-culture were separated using anti CD31 magnetic beads. Total RNA was isolated and specific mRNA levels were quantified by qRT PCR as described in “Methods”. The data are presented as fold changes in the mRNA levels of cells on Matrigel^TM^, in co-culture with MSCs, and proliferating/motile HUVECs compared to the mRNA levels for the same transcript in the confluent monolayer, “*”: *p* < 0.05, *n* ≥ 3.

Tip cells are different from stalk cells, forming the body of the newly formed vascular outgrowth. Tip-cell-enriched genes include PLAU, PLAUR, JAG1, DLL4, KDR, NRP1, EFNB2, ITGB1, PDGFB, ESM1, APLN, CCN1, and YAP1, and stalk-cell-enriched genes include DLL1, JAG1, and FLT1 (Figure 4a). Additionally, stalk cells have active Notch signaling accompanied with HES and HEY upregulation in contrast to tip cells, which have restricted Notch signaling [15,16,17].

Analysis of tip- or stalk-enriched genes did not show a clear picture. Most of tip and stalk genes were upregulated in HUVECs in the Matrigel^TM^ assay; however, APLN, ESM1, and CCN1 were downregulated. 

In the co-culture model, PLAU, PLAUR, and JAG1 were upregulated and DLL1, DLL4, EFNB2, and HEY1 were downregulated. In the proliferating/motile HUVECs, PLAU, PLAUR, JAG1, PDGFB, ESM1, APLN, and CCN1 were upregulated and DLL4, KDR, NRP1, EFNB2, ITGB1, YAP1, DLL1, FLT1, HES1, and HEY1 were downregulated (Figure 4b).

In Figure 1, we can see that HUVECs formed tip- and cell-like structure only in co-culture. On Matrigel^TM^, cells tended to form clusters (Figure 1b). 

### 3.4. Notch

**Figure 5:**
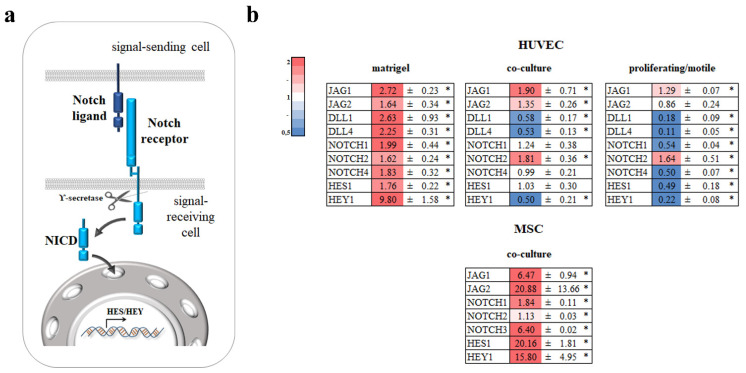
Analysis of Notch signaling genes. (**a**) Scheme illustrating Notch signaling. (**b**) HUVECs were grown on Matrigel^TM^, in co-culture with MSCs, or as proliferating/motile HUVECs or a confluent monolayer for 19 h. Cells in direct co-culture were separated using anti CD31 magnetic beads. Total RNA was isolated and specific mRNA levels were quantified by qRT PCR as described in “Methods”. The data are presented as fold changes in the mRNA levels of HUVECs on Matrigel^TM^, in co-culture with MSCs, proliferating/motile HUVECs compared to the mRNA levels for the same transcript in confluent monolayer, or to a monoculture for MSCs “*”: *p* < 0.05, *n* ≥ 3.

One of the important regulators of intracellular communication and cause of angiogenesis is Notch signaling (Figure 5a) [18]. We analyzed the expression of four Notch ligands: JAG1, JAG2, DLL1, and DLL4; three receptors: NOTCH1, NOTCH2, and NOTCH4; and transcription factors whose expression is initiated by the Notch signaling pathway: HES1 and HEY1. Interestingly, in HUVECs on Matrigel^TM^, all genes were upregulated. This indicates active Notch signaling. In proliferating/motile HUVECs, only JAG1 and NOTCH2 were upregulated and DLL1, DLL4, NOTCH1, NOTCH4, HES1, and HEY1 were downregulated, which indicates a decrease in the Notch signaling activity. In co-cultured HUVECs, JAG1, JAG2, and NOTCH2 were upregulated and DLL1, DLL4, and HEY1 were downregulated, which means a decrease in the Notch signaling activity. We also analyzed the expression of Notch genes in MSCs co-cultured with those HUVECs. In MSCs, JAG1, JAG2, NOTCH1, NOTCH2, NOTCH3, HES1, and HEY1 were upregulated. It is worth mentioning that JAG1 and NOTCH3 were upregulated more than 6-fold and JAG2 more than 20-fold (Figure 5b).

### 3.5. Ephrin–Eph Signaling Pathway

**Figure 6:**
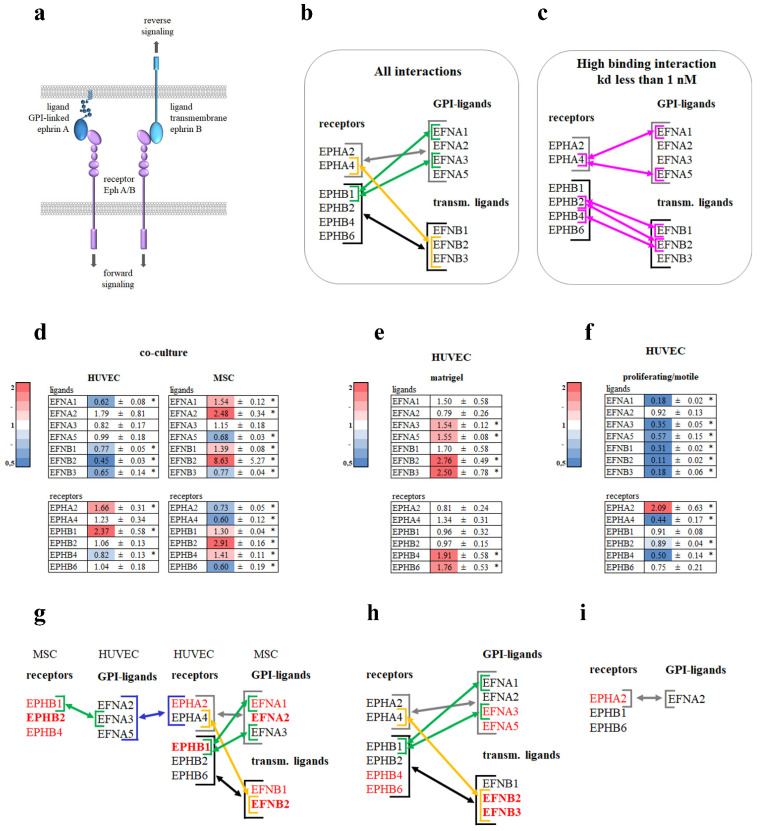
Analysis of EPH–EFN signaling genes. (**a**) Scheme illustrating EPH–EFN signaling. (**b**) Known interactions between EPH receptors and EFN ligands [21]. (**c**) High binding interactions, Kd <1 nM [19]. HUVECs were grown in co-culture with MSCs (**d**), on Matrigel^TM^ (**e**), proliferating/motile HUVECs (**f**), or a confluent monolayer for 19 h. Cells in direct co-culture were separated using anti CD31 magnetic beads. Total RNA was isolated and specific mRNA levels were quantified by qRT PCR as described in “Methods”. The data are presented as fold changes in the mRNA levels of HUVECs on Matrigel^TM^, in co-culture with MSCs, and proliferating/motile HUVECs compared to the mRNA levels for the same transcript in a confluent monolayer or to a monoculture for MSCs “*”: *p* < 0.05, *n* ≥ 3. Hypothetical scheme of EPH–EFN interactions in a co-culture of HUVECs and MSCs (**g**), HUVECs on Matrigel^TM^ (**h**), and proliferating/motile HUVECs (**i**). Bold font indicates fold of upregulation >2.3.

Another important angiogenic regulator that also works in tandem with Notch is the Ephrin–Eph signaling pathway (Figure 6a) [19,20]. 

EphA receptors mainly to bind ephrinA ligands and EphB receptors to ephrinB ligands; however, some receptor–ligand interactions also occur between opposite classes (Figure 6b) [19]. There are also high binding interactions (less than 1 nM) of ephrin ligands and EphB receptors within the same class [19] (Figure 6c).

We analyzed seven ephrin ligands: EFNA1, EFNA2, EFNA3, EFNA5, EFNB1, EFNB2, and EFNB3, and six ephrin receptors: EPHA2, EPHA4, EPHB1, EPHB2, EPHB4, and EPHB6. In HUVECs on Matrigel^TM^, no genes were downregulated and EFNA3, EFNA5, EFNB2, and EFNB3 were upregulated (Figure 6e). In proliferating/motile HUVECs, most genes were downregulated and only EPHA2 was upregulated (Figure 6f). In co-cultured HUVECs, EPHA2 and EFNB1 were upregulated and EFNA1, EFNB1, EFNB2, EFNB3, and EPHB4 were downregulated. In the corresponding co-cultured MSCs, EFNA1, EFNA2, EFNB1, EFNB2, EPHB1, EPHB2, and EPHB4 were upregulated and EFNA5, EFNB3, EPHA2, EPHA4, and EPHB6 were downregulated (Figure 6d).

We constructed a scheme of the proposed Ephrin–Eph interaction between HUVECs and MSCs in the co-culture model (Figure 6g), between HUVECs in the Matrigel^TM^ assay (Figure 6h), or between proliferating/motile HUVECs (Figure 6i) taking into account genes that were upregulated or changed insignificantly. 

The scheme of proliferating/motile HUVECs (Figure 6i) is the simplest and represents EPHA2–EFNA2 interaction. The richest pattern of interactions corresponds to HUVECs in the Matrigel^TM^ assay (Figure 6h).

### 3.6. Extracellular Matrix

Vessel growth is accompanied by extracellular matrix remodeling; the most significant step is basement membrane assembly [3].

**Figure 7:**
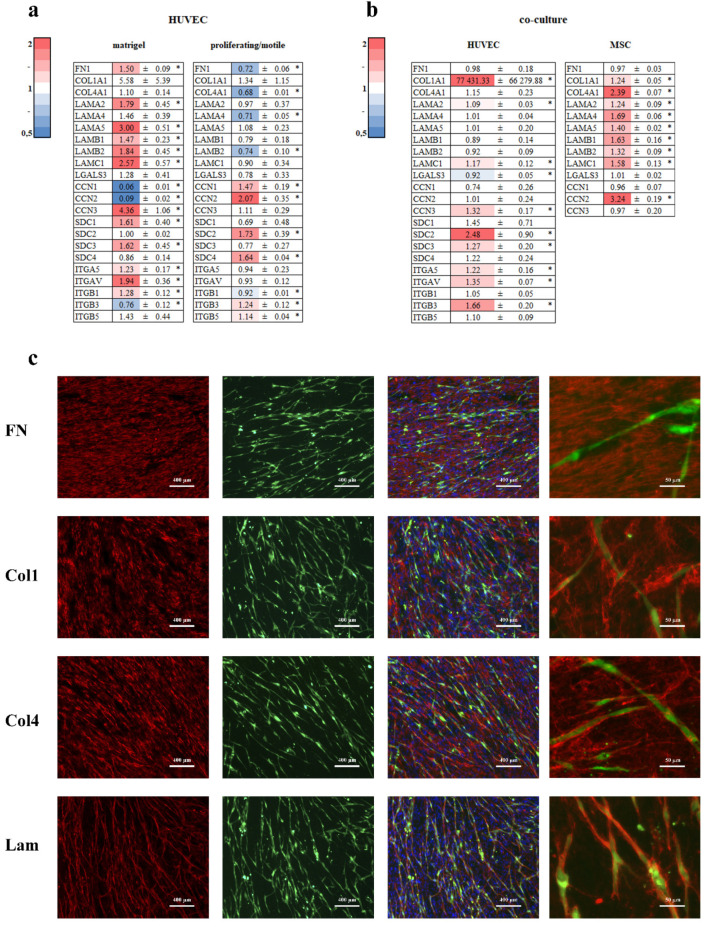
EM synthesis. HUVECs were grown on Matrigel^TM^ (**a**), as proliferating/motile HUVECs, in co-culture with MSCs (**b**), or as a confluent monolayer for 19 h. Cells in direct co-culture were separated using anti CD31 magnetic beads. Total RNA was isolated and specific mRNA levels were quantified by qRT PCR as described in “Methods”. The data are presented as fold changes in the mRNA levels of HUVECs on Matrigel^TM^, in co-culture with MSCs, proliferating/motile HUVECs compared to the mRNA levels for the same transcript in confluent monolayer, or to a monoculture for MSCs “*”: *p* < 0.05, *n* ≥ 3. (**c**) HUVECs were preloaded with CMFDA before being placed in direct co-culture. Cells were grown for 48 h, washed, fixed with 4% PFA, and stained with anti-fibronectin, collagen I, collagen IV, and laminin antibody conjugated with Alexa Fluor 594 (red). The nuclei were counterstained with DAPI (blue). Images were taken using an Axiovert 200 M microscope (Zeiss, Oberkochen, Germany).

We analyzed the mRNA expression of fibronectin, collagens, laminins, syndecans, CCN 1–3, galectin3 (LGALS3), and integrins in HUVECs on Matrigel^TM^, proliferating/motile HUVECs (Figure 7a), HUVECs, and MSCs from co-culture (Figure 7b). Proliferating/motile HUVECs in co-culture and in the Matrigel^TM^ assay were different in the extracellular matrix components expression profile.

In proliferating/motile HUVECs, extracellular matrix proteins FN1, COL4A1, LAMA4, and LAMB2 were downregulated and SDC2, SDC4, CCN1, and CCN2 were upregulated (Figure 7a). CCN1 and CCN2 upregulation may indicate turning off the Hippo pathway and this makes sense since cells lose contact and proliferate.

In HUVECs in the Matrigel^TM^ assay, FN1, LAMA2, LAMA5, LAMB1, LAMB2, LAMC1, SDC1, SDC3, CCN3, ITGA5, ITGAV, and ITGB1 were upregulated and only CCN1, CCN2, and ITGB3 were downregulated (Figure 7a). This means that HUVECs in the Matrigel^TM^ assay probably remodel the extracellular matrix by fibronectin synthesis and organization by integrin a5b1. The combination of upregulated laminin subunits indicates probable synthesis of basement membrane laminin 511 (a5b1c1). This poses many questions since Matrigel^TM^ is thought to be compositionally similar to the basement membrane. CCN1 and CCN2 downregulation may indicate an active Hippo pathway and it means that cells are in the final stage of ETN formation. 

In co-cultured HUVECs, LAMA2, LAMC1, SDC2, SDC3, CCN3, ITGA5, ITGAV, and ITGB3 were upregulated and only LGALS3 was slightly downregulated (Figure 7b). In co-cultured MSCs, COL1A1, COL4A1, LAMA2, LAMA4, LAMA5, LAMB1, LAMB2, and LAMC1 were upregulated. These data suggest MSCs contribute more to basement membrane synthesis than HUVECs. In Figure 7c, we can see that collagen1, collagen4, and laminin, the main components of the basement membrane, tended to cover tubular-like structures formed by HUVECs in co-culture with MSCs (Figure 7c) in contrast to fibronectin.

Next, we analyzed the impact of the extracellular matrix assembled by MSCs. Previously, we have shown that MSCs provide the basis for extracellular matrix assembly in co-culture with HUVECs [4]. We cultivated MSCs as a monoculture for 48 h (“48 hold MSCs”) to allow synthesis/secretion/assembly of the extracellular matrix. HUVECs were then seeded on top of the “48 h-old MSCs” and co-cultured for an additional 24 h. A comparison was made with the 24 h co-culture of HUVECs and MSCs (“0 hold MSCs”).

**Figure 8:**
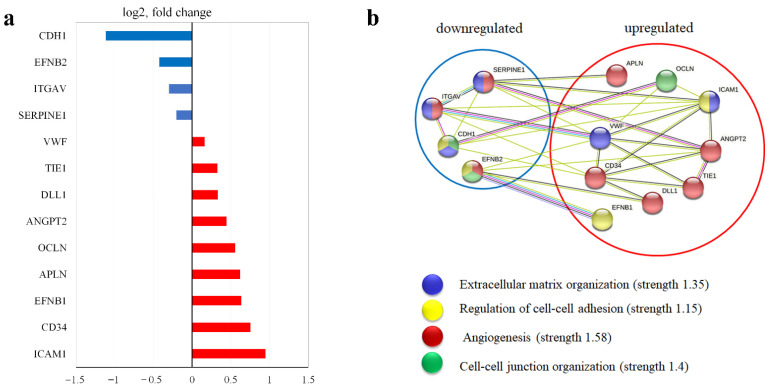
Input of EM synthesized by MSCs as a monoculture on gene expression of further co-culture with HUVECs. MSCs were grown as a monoculture for 48 h (“48 hold MSCs”) to allow synthesis/secretion/assembly of FN fibrils. HUVECs preloaded with CMFDA (green) were then seeded on top of “48 h-old MSCs” and co-cultured for an additional 19 h. Cells in direct co-culture were separated using anti-CD31 magnetic beads. Total RNA was isolated and specific mRNA levels were quantified by qRT PCR as described in “Methods”. The data are presented as fold changes in the mRNA levels of HUVECs in co-culture with “48 h-old MSCs” MSCs compared to the mRNA levels for the HUVECs in 19 h co-culture with “0 h-old MSCs”. (**a**) Fold changes in the expression significantly altered mRNA presented as log2. *p* < 0.05, *n* ≥ 3 experiments. (**b**) Functional protein association networks analysis was performed in STRING [9].

Pre-synthesized EM leads to the downregulation of CDH1, EFNB2, ITGAV, and SERPINE1 and upregulation of VWF, TIE1, DLL1, OCLN, APLN, EFNB1, ICAM1, and CCN2 (Figure 8a).

We analyzed these genes using functional protein association network analysis and attributed them to four groups with a strength of more than 1.15: extracellular matrix organization, regulation of cell–cell adhesion, angiogenesis, and cell–cell junction organization (Figure 8b). The upregulated gene set was enriched by angiogenesis genes.

### 3.7. Prep1

Homeodomain transcription factor PREP1 (PKNOX), which is thought to regulate angiogenesis through stimulation of EC migration, proliferation, and tube formation [22], was significantly upregulated in HUVECs in co-culture with MSCs (1.44 ± 0.27) and in proliferating/motile HUVECs (1.44 ± 0.13).

## 4. Discussion

We found that at a time point of 19 h, the tube formation on Matrigel^TM^ and tube formation in co-culture could be attributed to two different stages of angiogenesis. As a reference point, we chose a confluent monolayer. 

Hierarchical cluster analysis revealed that HUVECs in co-culture with MSCs and dividing/migrating HUVECs had more in common than HUVECs on Matrigel^TM^.

We found that although most of the analyzed genes were differentially expressed, some genes (PLAU, PLAUR, JAGd1, and NOTCH2) were upregulated in all experimental settings used. 

It is known that urokinase system members, uPA and uPAR, are attributed to EM remodeling and are expressed by activated endothelial cells but not in the quiescent vessels (or expressed at a low level) [23,24].

For Notch signaling, it is not all that clear-cut. In a growing vessel, in tip cells, Notch signaling is blocked while in stalk cells, it is active [25]. The suppression of Notch signaling in active angiogenesis leads to increased endothelial proliferation with excessive vascular branching [26]. Single-cell RNA sequencing in mice showed that Notch signaling is upregulated in mature capillary and arterial ECs [27]. At the same time, active Notch1 was detected by immunofluorescence in adult tissue only locally in vasculature, adipose tissue, kidney, brain, and lung [26]. Previously, we showed that inhibition of Notch signaling caused a decrease in the length of tube formation in a 2D co-culture model [4] and 3D co-culture model [28]. Here, we observed upregulation of Jagged1 and Notch2 in all three EC conditions.

The activation of Notch signaling in HUVECs in Matrigel^TM^ assay was accompanied by upregulation of all Notch ligands and receptors. In dividing/migrating HUVECs, the downregulation of receptors and ligands was accompanied by suppression of Notch signaling. Moreover, it looks like only Jagged1, Jagged2, and Notch2 were active in intercellular communication. A similar picture was observed for co-cultured ECs, but in the co-cultured MSCs, we observed activation of Notch signaling and strong upregulation of Notch ligands and receptors, excluding Notch2 (Figure 5). 

Intracellular interactions in co-culture are thought to be carried out through NOTCH1 and Jagged1 on ECs and NOTCH2, NOTCH3, and Jagged1 on vascular smooth muscle cells [29,30]. We found that Notch signaling in co-culture was performed mostly through Notch1/Notch3 on MSCs and Jagged1/Jagged2 on ECs (Figure 5). We can propose another important interaction through NOTCH2 on ECs and Jagged1/Jagged2 on MSCs. However, the intercellular domains of Notch1 and Notch2 are thought to be functionally equivalent [31] and the Notch2 extracellular domain appeared to be more efficient in inducing ligand-mediated receptor activation in kidney cells [32]. These data give a lot of food for thought and further experiments, especially in regard to Alagille syndrome, a dominant, multisystem disorder caused by mutations in the genes encoding Jagged1 and Notch2 [33], should be carried out. 

Others showed that JAG1 was upregulated in ECs at least 3 h after cells were plated on Matrigel^TM^ and remained upregulated at 6 h after being plated on Matrigel^TM^ [34].

Another process that goes hand in hand with Notch signaling is EPH-EFN signaling. EPH-EFN signaling is quite complex and intricate. It involves forward signaling through EPH receptors and reverse signaling through EFN ligands [19,20].

The EPHA2 receptor was upregulated and its ligand EFNA1 was downregulated in dividing/migrating HUVECs and in HUVECs in co-culture (Figure 6d,f). The results from other studies indicate that inversely to the expression of the GPI-ligand EFNA1, its receptor EPHA2 showed the highest expression at low cell densities [35]. As EFNA1 was downregulated, the only ligand that did not change its expression was EFNA2. Therefore, in dividing/migrating ECs, we can assume interaction occurs through EFNA2–EPHA2. 

The classical model of ECs–MSCs interaction involves EPHB4–EFNB2 [36].

Here, we observed an increase in EPHB1 expression in HUVECs in co-culture (Figure 6d). Others showed that EphB1 and EphA2 activation leads to an increase in cell migration and angiogenic sprouting [37]. These data agree with ours. However, the possibility of intracellular interaction of ECs with MSCs through ephrins requires additional research.

For HUVECs in the Matrigel^TM^ assay, the picture was much more complicated. No downregulation of EFN ligands or EPH receptors was observed. As EFNA1 and EPHA2 did not change compared to the confluent monolayer, it appears that HUVECs on Matrigel^TM^ reached a certain shape/structure. It is interesting that there was also upregulation of EPHB4 and EFNB2, which resembles the interactions of veins and arteries to stimulate remodeling and assembly of new vessels [38,39]. Of interest, Notch acts upstream of EPHB4/EFNB2 [26].

Figure 6g–i illustrates a hypothetical scheme of the EPH–EFN interaction of ECs in all three assays. 

We observed that HUVECs formed tip cells only in co-culture with MSCs (Figure 1b3,b4). However, alterations in gene expression indicated a possible tip–stalk pattern. In dividing/migrating HUVECs, we noticed downregulation of tip and stalk genes.

Activated endothelium loosens its contacts and tip cells undergo partial EndMT, becoming more motile. Key transcriptional regulators of EndMT are Snail (SNAI1), Slug (SNAI2), Zeb1 (ZEB1), Zeb2 (ZEB2), and Twist (TWIST1). The shared characteristic of these proteins is the transcription repression of E-cadherin (gene CDH1) [12]. However, these proteins differ in their functions and their influence on each other is very complicate. In cancer, Snail can induce endothelial-to-mesenchymal transition (EMT) in epithelial cells while Zeb1/2 and Twist maintain the invasive mesenchymal phenotype. Slug is the primary initiator of sprouting angiogenesis while the induction of Snail occurs at a much later time [12]. Snail can upregulate Zeb1 and Zeb2, Slug can activate Zeb1, and Twist1 can regulate the expression level of Snail and Slug. Slug and Snail negatively regulate each another’s expression [12]. Snail plays a major role in inducing EMT while Zeb1/2 and Twist are mainly involved in maintaining the invasive mesenchymal phenotype [12]. Slug-dependent pathways include regulators of cell morphology, junctional and matrix adhesions, proliferation, and TGFβ signaling [40].

ECs in co-culture with MSCs underwent EndMT to a greater extent than ECs on Matrigel^TM^. Surprisingly, dividing/migrating ECs did not undergo EndMT (Figure 3b). 

In this context, we should not forget about the different stiffness of Matrigel^TM^ and the plastic dish. 

Polystyrene has an elastic modulus of approximately 3 GPa. In comparison, the elastic modulus experienced by cells in situ in most tissues is four to six orders of magnitude lower [41]. The average modulus value for Matrigel^TM^ was found to be approximately 450 Pa for samples maintained at 37 °C [42]. Cells have been found to spread less on softer surfaces and those with a low density of EM protein at the surface while cells spread more on stiffer surfaces and those with a higher density of EM protein [43]. At a stiffness of 4 or 5 kPa of polymer, HUVECs spread as a monolayer. In contrast, at stiffness values between 0.5 and 1.5 kPa, HUVECs formed a tubular network comparable with that on the Matrigel^TM^ [44]. The blocking of laminin and integrins α1, α2, α3, and α6 inhibits tube formation and cells form an endothelial monolayer [44], underling the role of laminins in tubular network formation.

Previously, we have shown that HUVECs placed on MSCs that were pre-grown for 48 h and synthesized an EM scaffold formed ETN faster than those placed into a co-culture with MSCs on plastic [4].

However, we found that extracellular matrix pre-synthesized by MSCs as a monoculture was adequate for ECs to create ETN similar to on Matrigel^TM^ [4]. 

We think that in the absence of endothelial cells, MSCs do not form elements of the basement membrane as part of the extracellular matrix. The major laminin isoforms of the vascular BM are laminin-411 (α4:β1:γ1) and laminin-511 (α5:β1:γ1) [45]. According to the PCR data, MSCs contributed the most to laminin deposition (Figure 7b) but only in co-culture with ECs (Figure 7b). As for collagen type IV, Yamamoto showed that in HUVEC or MSC monocultures, collagen type IV deposition was not detected and was deposited around capillary structures covered by MSC-derived pericytes [46]. It is supposed that in the absence of mural cells, ECs could not also form the basement membrane. Both cell types, ECs and MSCs, are involved in endothelial basement membrane assembly in co-culture.

This prompted us to switch to the idea of EC interplay with the basement membrane. In Matrigel^TM^ assay, it represents the basement membrane matrix by itself [3] while in the co-culture model, basement membrane deposition is limited to only the cell-to-cell contact area. 

It was shown that mature vessel formation accomplished with basement membrane formation leads to the arrest of division, migration, and stabilization of the vessel structure [47,48].

Other EM proteins include syndecans. The syndecan family consists of syndecan-1, syndecan-2, syndecan-2, and syndecan-4 [49]. These proteins act like molecular glue to stabilize signaling complexes on the cell surface (including integrins, growth factors, and others), acting as co-receptors to modify the activity of integrins and interact with heparin-binding domains in extracellular matrix molecules and growth factors. Syndecan-1 is a key regulator of angiogenesis and syndecan-2 impairs angiogenesis [49]. Syndecan-1 is thought to be involved in EMT, syndecan-2 in angiogenesis, syndecan-3 in mitogenic signal transduction, and syndecan-4 in the formation of focal contacts and in the shear stress-sensing complexis [50]. Our data indicate that syndecan-4 was upregulated only in ECs in the co-culture model. However, at the same time, syndecan-2, which reduces angiogenesis, was upregulated in dividing/migrating ECs and in ECs in co-culture. This controversial data needs additional experiments.

The CCN1 (CYR61) protein and the CCN2 protein (CTGF) are positive regulators of angiogenesis through the engagement of integrin αvβ3. The CCN3 (NOV) protein displays a divergent function, providing protection from aberrant excessive vessel growth [49]. Downregulation of the CCN1 and CCN2 Hippo target genes in ECs on Matrigel^TM^ suggests activation of the Hippo signaling pathway while in dividing/migrating ECs, this pathway was probably inactive. Taking this into account, we can conclude that ECs in Matrigel^TM^ assay at 19 h display the final stage of tube formation, with upregulation of CCN3 and downregulation of CCN1 and CCN2 (Figure 7a). 

Others showed that CCN1 was downregulated in HUVECs at least 3 h after being placed on the Matrigel^TM^, the same time point that ECs began forming tubes [34]. For ECs in co-culture, 18 h is the time that ECs began forming tubes [4]. 

**Figure 9:**
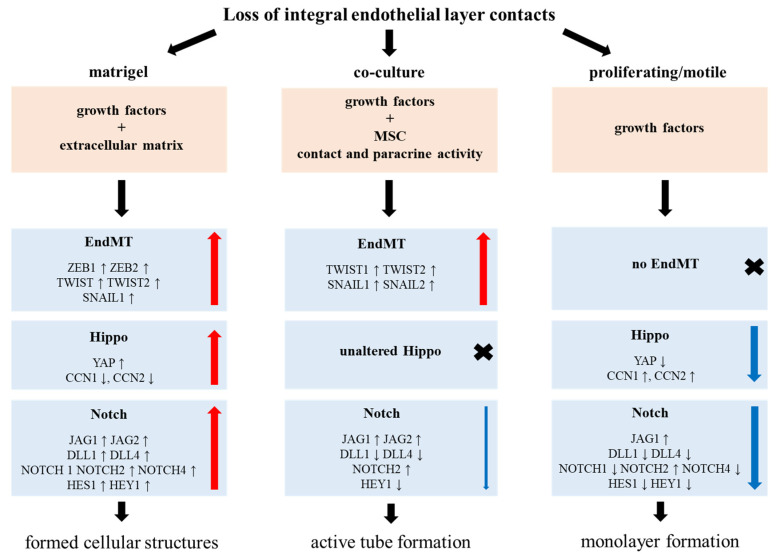
Hypothetical scheme illustrating processes occurring in HUVECs grown on Matrigel^TM^, in co-culture with MSCs, and proliferating/motile HUVECs. Red arrows indicate expression level increase, blue arrows indicate expression level decrease. Arrows thickness indicates the intensity of the expression change.

Therefore, we suggest that HUVECs in co-culture and proliferating/motile ECs are still in a dynamic process at 19 h. In contrast, HUVECs in the Matrigel^TM^ assay seem to be in the final stage of ETN formation due to the fully pre-assembled basement membrane (Matrigel^TM^ itself) (Figure 9).

We can conclude that the mechanisms underlying ETN formation in these models are completely different due to ECs–EM interplay. 

The final stage of endothelium stabilization can be monitored by endothelium activation factors, for example, ANGPT2, VCAM1, ICAM-1, and MCP1. ANGPT1 is expressed in mural cells as an agonistic ligand for the angiopoietin 1 receptor (TIE2) expressed on the surface of ECs whereas ANGPT2 is predominantly expressed as an antagonistic ligand for TIE2 and stored by ECs [51]. During inflammation or hypoxia, ECs deactivate the quiescence signal of TIE2 by expressing ANG2, which competes with ANGT1 for binding to TIE2. This leads to a loss of TIE2 signaling and activation of the endothelium by weakening endothelial cell–cell junctions and induction of the expression of the pro-inflammatory adhesion molecules, ICAM1 and VCAM1 [51]. VCAM1 and ICAM1 are also known to be upregulated in low, oscillatory flow conditions [52].

We observed upregulation of ANGPT2 and VCAM1 and ICAM-1 and the same downregulation of MCP1 in ECs on Matrigel^TM^ (Figure 3b). In dividing/migrating ECs and in ECs in co-culture, VCAM1 was downregulated. On the one hand, this means that ECs on Matrigel^TM^ became activated, but on the other hand, they could already complete the process of tubular network formation. 

Other works showed an increase in VCAM1 as early as 1 h after the beginning of tube formation in the Matrigel^TM^ assay in human microvascular endothelial cells (HMVECs) [53]. This indicates that this process is very quick. 

Other arguments in support of our hypothesis regarding the Matrigel^TM^ assay as a representation of the final stage of tissue formation are:

1. Apelin, which is thought to promote cell growth [54], was downregulated in ECs on Matrigel^TM^ and was upregulated in dividing/migrating ECs.
2. ESM-1 (endocan) is associated with filopodia in tip cells [15]. It was downregulated in ECs on Matrigel^TM^ and upregulated in dividing/migrating ECs.
3. EDN1 is an autocrine stimulus of EC proliferation and migration [55] and was downregulated in ECs on Matrigel^TM^ and upregulated in dividing/migrating ECs.
4. Junction proteins TJP1 and OCLN were upregulated in ECs on Matrigel^TM^ and downregulated in ECs in co-culture and in dividing/migrating ECs.
5. TIE2 (TEK), which provides endothelial quiescence, was upregulated in ECs on Matrigel^TM^ and downregulated in ECs in co-culture.

Thirty-four years ago, Kubota et al. had already shown that ECs in tubular-like structures on Matrigel^TM^ did not proliferate, formed tight cell–cell contacts, and took up acetylated LDL, which is a marker of differentiation for these cells, and this was not observed for cells cultured in a monolayer on either plastic or collagen I substrates [1]. Moreover, a higher cell density leads to more concentrated clusters but not to greater tube formation [56]. 

The above-mentioned evidence and our new data indicate that EC tube formation in the Matrigel^TM^ assay and in a co-culture model rely on quite different processes despite being morphologically similar. We suppose that ECs in the Matrigel^TM^ assay are trying to set up the vessel lining, spreading over the basement membrane (Matrigel^TM^). ECs form tight contacts and attach to Matrigel^TM^ through laminin receptors that prevent monolayer formation, as in the case of more rigid surfaces such as plastic dishes.

Differences in the expression levels of key genes involved in the EndMT, Hippo, and Notch auxiliary angiogenic pathways are presented in a summarizing scheme (Figure 9).

On the other hand, in the MSC co-culture model, ECs form tubular-like structures through interaction with stromal cells that alters both the cell expression profiles and causes basement membrane assembly. ECs, in this case, form ETN only in places covered by basement membrane proteins such as laminins and collagen IV.

One particularly interesting finding of our work was the absence of EndMT in dividing/migrating ECs, but at the same time, they had upregulated tip cell markers (PLAU, PLAUR, ESM1, APLN, CCN1) and decreased Notch signaling. This unexpected combination of altered genes at this time point requires additional comprehensive research.

Undoubtedly, one cell type cannot approximate the behavior of endothelial cells from other organs and tissues. In this work, we used HUVECs because of several benefits. HUVECs are the most commonly used type of endothelial cells in the published research on biomaterials. HUVECs are easy to isolate without contamination by other cell types, and umbilical cord is readily available as a discarded biological waste after childbirth. In contrast, the isolation of human microvascular endothelial cells has a number of disadvantages, such as the small isolation yield, admixture of mesenchyme-like cells, and low proliferative activity. In simple 2D in vitro models, HUVECs and microvascular endothelial cells show a similar response to external stimuli. Sieminski et al. showed that HUVECs and human dermal microvascular endothelial cells have much in common in simple in vitro models: vascular network formation, gel contraction, cell elongation, survival, and inhibition of network formation by blocking antibodies to a2b1 but not avb3 integrins [57]. Lidington et al. showed that HUVECs and the immortal human endothelial cell line HMEC-1 (human dermal microvascular endothelial cells) have a similar pattern of expression for PECAM-1, ICAM-1, and VCAM-1 [58].

However, despite all their benefits, HUVECs do not necessarily represent a universal model of endothelial cells for every application. For more complex 3D models of angiogenesis or for the production of a functional vasculature within in vitro engineered tissues, whether the endothelial cells belong to microvessels or large vessels is more crucial.

We hope that our results will provide new insights for the understanding of angiogenesis mechanisms.

## Figures and Tables

**Table 1:** List of PCR primers used.

Gene Name	Forward	Reverse
ACTB	cctggcacccagcacaat	gggccggactcgtcatac
ACTA2	aagaggaatcctgaccctgaa	gccagatcttttccatgtcg
APLN	gtctcctccatagattggtctgc	ggaatcatccaaactacagccag
CAV1	catcccgatggcactcatctg	tgcactgaatctcaatcaggaag
CCL2	cagccagatgcaatcaatgcc	tggaatcctgaacccacttct
CCN1	ccctcgcggcttaccgactgg	cacaggtcttggaacaggcgc
CCN3	aactgcattgaacagaccaca	attgacggttcctattggtgac
CDH1	atttttccctcgacacccgat	tcccaggcgtagaccaaga
CDH2	agccaaccttaactgaggagt	ggcaagttgattggagggatg
CDH5	acgcctctgtcatgtaccaa	acgatctcatacctggcctg
CDH13	agtgttccatatcaatcagccag	cgagacctcatagcgtagctt
CLDN5	ctctgctggttcgccaacat	cagctcgtacttctgcgaca
COL1A1	gagggccaagacgaagacatc	cagatcacgtcatcgcacaac
COL4A1	ggcatgcctggtattggt	aggccccatatcacccttag
DLL1	tgtgacgagtgtatccgctat	gtgtgcagtagttcaggtcct
DLL4	gcactccctggcaatgtact	gtggtgggtgcagtagttga
EDN1	agagtgtgtctacttctgcca	cttccaagtccatacggaacaa
EFNA2	ctccctgggcttcgagtt	ccgcacgtacaccttcagt
EFNA5	cactcccttttctctaggatttga	gacaggaccttcttccattatctg
EFNB1	tcatgaaggttgggcaaga	cagtgttgtctgcctccttg
EFNB3	tggaactcggcgaataagag	cgatctgagggtacagcaca
EFNA1	aacaagctgtgcaggcatgg	ctccacagatgaggtcttgc
EFNA3	actctcccccagttcacca	gcacctgagggttctctcc
EFNB2	tctttggagggcctggat	ccagcagaacttgcatcttg
EPHA2	ccaggcaggctacgagaa	ggctctcagatgcctcaaac
EPHA4	catgtcccgagtgcttgag	cagtccaccggataggaatc
EPHB1	tggctatgagcctgagaacag	agtgggagcagccttcag
EPHB2	tgtctcagatgatgatggagga	ccgcatcacctggatactgt
EPHB4	cggatcctacccgagtga	tgtgttcagcagggtctcttc
EPHB6	tcctcgaatggcagaaaaag	ttctgcaaggggttattcca
ESM1	cagggggacgggaaaatgc	cagatgccatgtcatgctcc
FLT1	cttatgatgccagcaagtgg	caaaagcccctcttccaagt
FN1	gacgcatcacttgcacttct	gcaggtttcctcgattatcct
ITGA5	ggcttcaacttagacgcggag	tggctggtattagccttgggt
ITGAV	attctgtggctgtcggagat	ccttgctgctcttggaactc
ITGB1	ttctccagaaggtggtttcg	acaccagcagccgtgtaac
ITGB3	cagcccgggtcatctgta	agctctccctgactcctcct
ITGB5	ggcttcaacttagacgcggag	tggctggtattagccttgggt
JAG1	tcgctgtatctgtccacctg	agtcactggcacggttgtag
JAG2	tgggactgggacaacgatac	agtggcgctgtagtagttctc
KDR	ccttccagaccgttacgtg	agccagagctgcatcatttc
LAMA2	ggcttattcagctggcagag	attctcccagggactttgct
LAMA4	gactgccaacagtgccaac	ccaccctgataggtgccat
LAMA5	ggatgccgaagacatgaac	ttccctcactctttcctgttgt
LAMB1	aacgtggttggaagaacctg	acactccctggaaacagtgg
LAMB2	ggacgaaaagaagtgcttcct	gcagggataccattctctgact
LAMC1	gagttcgtcaacgccgc	cctggttgttgtagtcggtca
MMP14	atcatggcacccttttacca	gcgctccttgaagacaaac
NOTCH1	cggggctaacaaagatatgc	caccttggcggtctcgta
NOTCH2	tggtggcagaactgatcaac	ctgcccagtgaagagcagat
NOTCH3	cctagtcctggctccgaac	gagccggttgtcaatctcc
NOTCH4	gatgggctggacacctacac	cacacgcagtgaaagctacca
NRP1	acgtggaagtcttcgatggag	caccatgtgtttcgtagtcaga
OCLN	acaagcggttttatccagagtc	gtcatccacaggcgaagttaat
SERPINE1	ccagctgacaacaggaggag	cccatgagctccttgtacagat
PDGFB	tcccgaggagctttatgaga	gggtcatgttcaggtccaac
PECAM1	aacagtgttgacatgaagagcc	tgtaaaacagcacgtcatcctt
PKNOX1	atagacagctatcaagatgggca	gcatcgggttcagagcagttt
S100A4	gatgagcaacttggacagcaa	ctgggctgcttatctgggaag
SDC1	acggctattcccacgtctc	tctggcaggactacagcctc
SDC2	tcattgctggtggagttattgg	agcactggatggtttgcgt
SDC3	tggcgcagtgagaacttcg	ccccgagtagaggtcatccag
SDC4	tccccaccgaacccaagaa	ccttgttggacacatcctcac
SNAI1	tcggaagcctaactacagcga	agatgagcattggcagcgag
SNAI2	cgaactggacacacatacagtg	ctgaggatctctggttgtggt
TEK	tccgctggaagttactcaaga	gaactcgcccttcacagaaataa
TGFB1	cgctgcccatcgtgtacta	cgcacgatcatgttggac
TJP1	ctggtgaaatcccggaaaaatga	ttgctgccaaactatcttgtga
TIE1	aagcagacagacgtgatctgg	gcacgatgagccgaaagaag
TWIST1	gtccgcagtcttacgaggag	gcttgagggtctgaatcttgct
TWIST2	gcaagtggaattgggatgc	tcttctgtccgatgtcactgc
PLAU	tcaaaaacctgctatgagggga	gggcatggtacgtttgctg
PLAUR	tattcccgaagccgttacctc	ggtggcggtcatcctttgg
VCAM1	attcaaactgccctgatggg	ggtaaggttcttgcccactg
ICAM1	ataatgggaatctacagcacct	aacatgactgagtctccaatctg
VIM	agtccactgagtaccggagac	catttcacgcatctggcgttc
VWF	ccgatgcagccttttcgga	tccccaagatacacggagagg
YAP1	tagccctgcgtagccagtta	tcatgcttagtccactgtctgt
ZEB1	ttacacctttgcatacagaaccc	tttacgattacacccagactgc
ZEB2	gcgatggtcatgcagtcag	caggtggcaggtcattttctt
CCN2	ccctcgcggcttaccgactgg	cacaggtcttggaacaggcgc
LGALS3	cttataacctgcctttgcctgg	gcaacatcattccctctttgga
ANGPT1	tcgtgagagtacgacagacca	tctccgacttcatgttttccac
ANGPT2	accccactgttgctaaagaaga	ccatcctcacgtcgctgaata
CD34	ctacaacacctagtacccttgga	ggtgaacactgtgctgattaca
HES1	gaagcacctccggaacct	gtcacctcgttcatgcactc
HEY1	catacggcaggagggaaag	gcatctagtccttcaatgatgct

**Table 2:** Genes and corresponding proteins.

Gene Name	Decoding Gene Name	Protein Name	Decoding Protein Name
ACTA2	Actin Alpha 2, Smooth Muscle	α-SMA	Actin Alpha 2, Smooth Muscle
ANGPT1	Angiopoietin 1	Angiopoietin 1	
ANGPT2	Angiopoietin 2	Angiopoietin 2	
APLN	Apelin	Apelin	
CAV1	Caveolin 1	Caveolin	
CCL2	C-C Motif Chemokine Ligand 2	MCP-1	Monocyte Chemoattractant Protein 1
CCN1	Cellular Communication Network Factor 1	CYR61	CYsteine-Rich angiogenic inducer 61
CCN2	Cellular Communication Network Factor 2	CTGF	Connective Tissue Growth Factor
CCN3	Cellular Communication Network Factor 3	NOV	Nephroblastoma OVerexpressed protein
CD34		CD34 molecule	
CDH1	Cadherin 1	E-cadherin	Epithelial cadherin
CDH13	Cadherin 13	T-cadherin/ H-cadherin	Tranceted cadherin/Heart cadherin
CDH2	Cadherin 2	N-cadherin	Neural cadherin
CDH5	Cadherin 5	VE-cadherin	Vascular Endothelial cadherin
CLDN5	Claudin-5	Claudin-5	
COL1A1	Collagen type I alpha 1 chain	collagen type I alpha 1 chain	
COL4A1	Collagen type IV alpha 1 chain	collagen type IV alpha 1 chain	
DLL1	Delta Like canonical Notch Ligand 1	DLL1	Delta Like canonical Notch Ligand 1
DLL4	Delta Like canonical Notch Ligand 4	DLL4	Delta Like canonical Notch Ligand 4
EDN1	Endothelin 1	ET-1	endothelin 1
EFNA1	ligand ephrin A1	ephrin A1	ligand ephrin A1
EFNA2	ligand ephrin A2	ephrin A2	ligand ephrin A2
EFNA3	ligand ephrin A3	ephrin A5	ligand ephrin A3
EFNA5	ligand ephrin A5	ephrin A4	ligand ephrin A5
EFNB1	ligand ephrin B1	ephrin B1	ligand ephrin B1
EFNB2	ligand ephrin B2	ephrin B2	ligand ephrin B2
EFNB3	ligand ephrin B3	ephrin B3	ligand ephrin B3
EPHA2	EPH receptor A2	EPH-A2	EPH receptor A2
EPHA4	EPH receptor A4	EPH-A4	EPH receptor A4
EPHB1	EPH receptor B1	EPH-B1	EPH receptor B1
EPHB2	EPH receptor B2	EPH-B2	EPH receptor B2
EPHB4	EPH receptor B4	EPH-B4	EPH receptor B4
EPHB6	EPH receptor B6	EPH-B6	EPH receptor B6
ESM1	Endothelial cell Specific Molecule 1	endocan	
FLT1	Fms Related Receptor Tyrosine Kinase 1	VEGFR1	Vascular Endothelial Growth Factor Receptor 1
FN1	Fibronectin 1	fibronectin	
HES1	Hes Family BHLH Transcription Factor 1	HES1	Hes Family BHLH Transcription Factor 1
HEY1	Hes Related Family BHLH Transcription Factor With YRPW Motif 1	HEY1	Hes Related Family BHLH Transcription Factor With YRPW Motif 1
ICAM1	InterCellular Adhesion Molecule 1	ICAM1	InterCellular Adhesion Molecule 1
ITGA5	Integrin Subunit Alpha 5	integrin alpha 5/CD49e	integrin subunit alpha 5
ITGAV	Integrin Subunit Alpha V	integrin alpha V/CD51	integrin subunit alpha V
ITGB1	Integrin Subunit Beta 1	integrin beta 1/CD29	integrin subunit beta 1
ITGB3	Integrin Subunit Beta 3	integrin beta 3/CD61	integrin subunit beta 3
ITGB5	Integrin Subunit Beta 5	integrin beta 5	integrin subunit beta 5
JAG1	Jagged canonical Notch ligand 1	Jagged1	Jagged canonical Notch ligand 1
JAG2	Jagged canonical Notch ligand 2	Jagged2	Jagged canonical Notch ligand 2
KDR	Kinase Insert Domain Receptor	VEGFR-2	Vascular Endothelial Growth Factor Receptor 2
LAMA2	Laminin subunit alpha-2	laminin subunit alpha-2	
LAMA4	Laminin subunit alpha-4	laminin subunit alpha-4	
LAMA5	Laminin subunit alpha-5	laminin subunit alpha-5	
LAMB1	Laminin subunit beta-1	laminin subunit beta-1	
LAMB2	Laminin subunit beta-1	laminin subunit beta-1	
LAMC1	Laminin subunit gamma-1	laminin subunit gamma-1	
LGALS3	Galectin-3	galectin-3	
MMP14	Matrix MetalloProteinase-14	MMP14	Matrix MetalloProteinase-14
NOTCH1	Notch Receptor 1	Notch 1	Notch Receptor 1
NOTCH2	Notch Receptor 2	Notch 2	Notch Receptor 2
NOTCH3	Notch Receptor 3	Notch 3	Notch Receptor 3
NOTCH4	Notch Receptor 4	Notch 4	Notch Receptor 4
NRP1	Neuropilin-1	Neuropilin-1	
OCLN	Occludin	Occludin	
PDGFB	Platelet-derived growth factor subunit B`	Platelet-derived growth factor subunit B	
PECAM1	Platelet endothelial cell adhesion molecule 1	CD31	Cluster of Differentiation 31
PKNOX1	PBX/Knotted 1 Homeobox 1	PREP1	Pbx Regulating Protein-1
PLAU	Plasminogen Activator, Urokinase	Urokinase/uPA	urokinase-type plasminogen activator
PLAUR	Plasminogen Activator, Urokinase Receptor	urokinase receptor/uPAR/CD 87	urokinase plasminogen activator surface receptor/Cluster of Differentiation 87
S100A4	S100 calcium binding protein A4	FSP1	Fibroblast-Specific Protein 1
SDC1	Syndecan 1	syndecan 1	
SDC2	Syndecan 2	syndecan 2	
SDC3	Syndecan 3	syndecan 3	
SDC4	Syndecan 4	syndecan 4	
SERPINE1	Serpin family E member 1	PAI-1	Plasminogen Activator Inhibitor-1
SNAI1	Snail Family Transcriptional Repressor 1	SNAIL	
SNAI2	Snail Family Transcriptional Repressor 2	SLUG	
TEK	Tunica interna Endothelial cell Kinase	TIE 2/Angiopoietin-1 receptor	Tyrosine Kinase With Ig And EGF Homology Domains-2
TGFB1	Transforming Growth Factor beta 1	TGF-b1	Transforming Growth Factor beta 1
TIE1	Tyrosine Kinase With Immunoglobulin Like And EGF Like Domains 1	TIE1Angiopoietin-2 receptor	
TJP1	Tight Junction Protein 1	TJP-1/ZO-1	Tight junction protein-1/Zonula occludens-1
TWIST1	Twist Family BHLH Transcription Factor 1	TWIST1	Twist Family BHLH Transcription Factor 1
TWIST2	Twist Family BHLH Transcription Factor 2	TWIST2	Twist Family BHLH Transcription Factor 2
VCAM-1	Vascular Cell Adhesion Molecule 1	VCAM-1/CD106	Vascular Cell Adhesion Molecule 1/Cluster of Differentiation 106
VIM	Vimentin	vimentin	
VWF	Von Willebrand Factor	VWF	Von Willebrand Factor
YAP1	Yes1 Associated Transcriptional Regulator	YAP1	Yes1 Associated transcriptional regulator
ZEB1	Zinc finger E-box Binding homeobox 1	ZEB1	Zinc finger E-box Binding homeobox 1
ZEB2	Zinc finger E-box Binding homeobox 2	ZEB2	Zinc finger E-box Binding homeobox 2

## Data Availability

Not applicable.

## Abbreviations

BM	basement membrane
EC	endothelial cell
EM	extracellular matrix
EMT	epithelial-to-mesenchymal transition
EndMT	endothelial-to-mesenchymal transition
ETN	endothelial tubular network
GF	growth factors
HUVEC	human umbilical vein endothelial cell
MSC	mesenchymal stromal cell
